# Liabilities of the Muscle in Disease

**Published:** 1846-07

**Authors:** 


*Liabilities of the muscle in Disease.* Of epidemic influences that disturb the general health, the voluntary muscles take early and constant notice; for of life, in all its varieties of action, they are the truest, readiest, and most delicate exponents. Rheumatism, influenza, diarrhœa, illness of whatever kind that is "going about," prevail, by acknowledged symptoms, in the locomotive structures of the body. All the animal blights, electrical, contagious, miasmatic, are necessarily muscular in their development. Observe your patient, then, from first to last, as he stands, walks, sits, or lies; note well his changes of posture; see what he does with his hands; watch his features at their several periods of action and repose; compare them in their separate play. Nothing, be assured, is more truly clinical than such indication, by impaired contractility of disorder in the flesh. Remember, without disparagement of the medicine that works by dissection, analysis, and the microscope, that while engaged in the contemplation of these muscular symptoms, we have before us nothing less than the actual visible operations of disease. Observing them, we have under our wide, natural eye, not the mere segments of perverted, fast-decaying structures, not the shadowy, lenticular, spectra of a discharged and damaged fluid, but organs, living and complete, in active relation, through their function, with the blood and all else that is vital in the body. Here, in the Queen's ward, is a woman, (Mary Mc B——,) who tells us plainly, though not in words, of fast improvement and recovery. Near-sighted as I am, I see already, as we approach her bed, that since yesterday she is better. I see it, and at once, in the shape of her features; I know it by the very "wag of her eyelid." In this case, the buccinator and the levator palpebræ muscles express as much of encouragement as could be spoken by the mouth and larynx. Try and remember this patient as we knew her on March 11, when she was first admitted, scarcely conscious, exhausted, inarticulate,—how she lay, and whined, and stared. Day after day we found her stretched, as if by palsy, on her back; her knees were never bent; her hands moved but seldom from her side. In this unnatural repose of all the voluntary muscles, we could not fail to recognise the character and intensity of the disorder. The influence that operated to the prejudice of the contractile function, was, in this instance, atmospheric, and of the season. It is a case, now convalescent, of the spotted epidemic fever.—*London Lancet.*

